# Identification and characterization of cancer stem cells in canine mammary tumors

**DOI:** 10.1186/s13028-016-0268-6

**Published:** 2016-12-19

**Authors:** Agata Rybicka, Magdalena Król

**Affiliations:** Department of Physiological Sciences, Faculty of Veterinary Medicine, Warsaw University of Life Sciences, Warsaw, Poland

**Keywords:** Cancer stem cells, Canine mammary tumor, Translational medicine

## Abstract

Cancer stem cells (CSC) represent a small subpopulation of cells in malignant tumors that possess the unique ability to self-renew, differentiate and resist chemo- and radiotherapy. These cells have been postulated to be the basis for some of the difficulties in treating cancer, and therefore, numerous approaches have been developed to specifically target and eliminate CSC in diverse types of cancer, including breast cancer. Spontaneously occurring mammary tumors in canines share clinical and molecular similarities with the human counterpart, making the dog a potentially powerful model for the study of human breast cancer and clinical trials. Studies focused on canine mammary CSC might therefore enhance our understanding of the biology and possible treatment of the disease in both dogs and humans. In this review, we discuss various approaches currently in use to isolate and characterize canine mammary CSC.

## Background

Canine mammary tumors (CMT) remain some of the most common neoplasms affecting female dogs. Approximately 200–250 new diagnoses of CMT per 100,000 bitches are reported annually [1, 2], and of these cases, ~50% are malignant [3, 4]. Surgical removal of the tumor is a standard treatment for CMT, but in many cases, the procedure alone is ineffective due to existing micro-metastases [3]. Moreover, lack of effective chemo- and radiotherapy following mastectomy excludes possible alternative treatment strategies.

Recently, it has been hypothesized that a small subpopulation of cells, cancer stem cells (CSC), underlies tumor initiation, progression and metastasis. CSC are thought to share similar properties with normal stem cells, such as the ability to self-renew and differentiate into various cell types. High expression of adenosine triphosphate (ATP)-binding cassette transporters, quiescence and specific DNA repair abilities also make them remarkably drug resistant [5]. Therefore, understanding the biology of CSC and critical pathways involved in their maintenance might lead us to the discovery of novel compounds for breast cancer therapy.

Interestingly, CMT shares many clinical and molecular similarities with human breast cancer. Epidemiology and tumor behaviour follow the same pattern as in humans, rendering spontaneously occurring CMT an interesting model for breast cancer preclinical studies. Thus, close cooperation between human and veterinary medicine in breast cancer studies is likely to be of mutual benefit for treatment of the disease in both humans and dogs.

This review summarizes current knowledge regarding canine mammary CSC. Various techniques used for CSC isolation, the signaling pathways involved in tumor development, and studies regarding drug resistance and targeted treatment are presented. In addition, current challenges and future perspectives in the study of CMT are discussed.

## Search strategy

A literature search was performed in PubMed (http://www.ncbi.nlm.nih.gov/pubmed) using the terms “canine mammary tumor” OR “canine neoplasms” AND “cancer stem cells” OR “canine mammary cancer stem cells”. Only papers written in English and published within the last 25 years through November 2016 were included. All articles based on cancer stem-like cells isolated from tumor samples and canine mammary tumor cell lines were incorporated. Studies employing immunohistochemistry as a method for the identification of cancer stem-like cells in situ were evaluated, and the most relevant to our review were selected. An additional resource was our own data from the isolation and identification of canine cancer stem-like cells [6, 7].

## Canine model for breast cancer studies

**Fig. 1:**
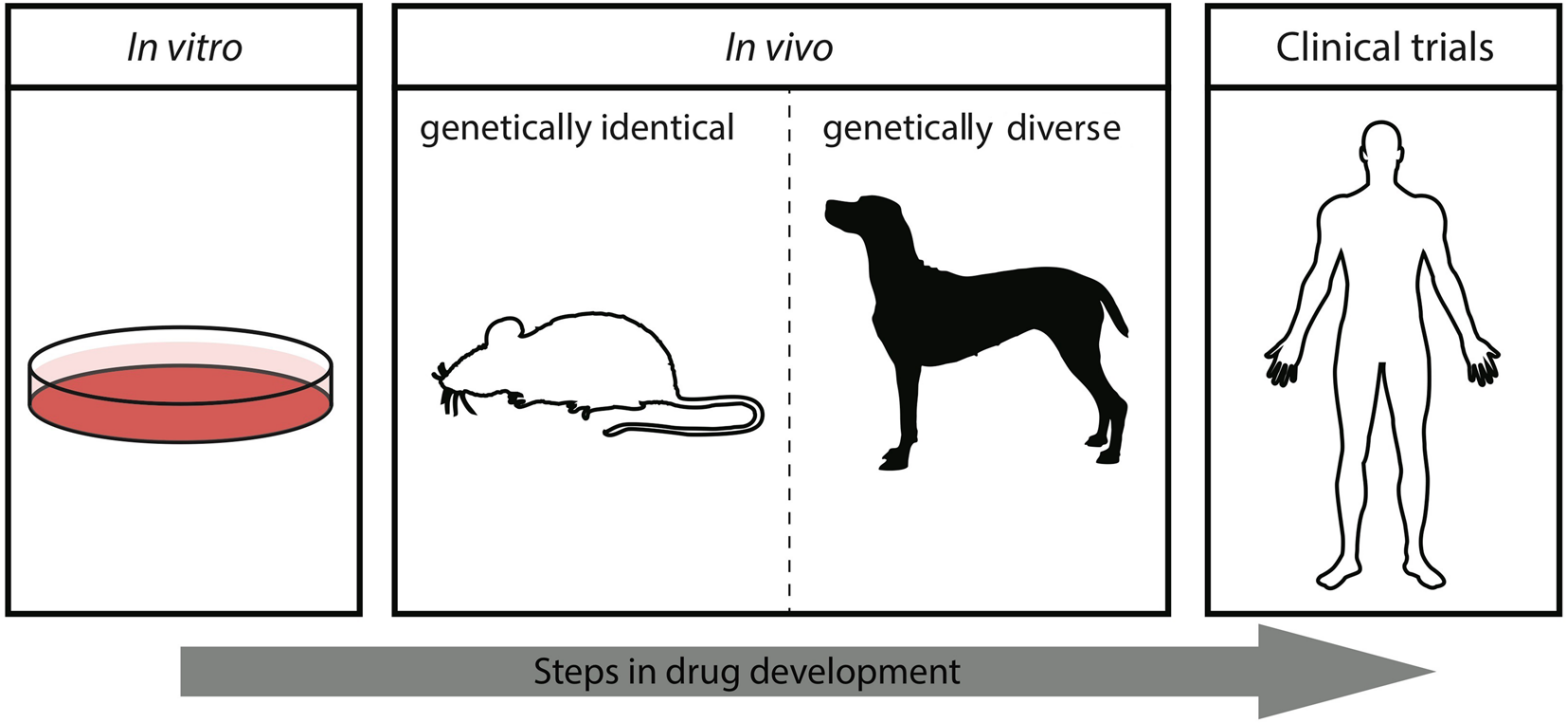
The canine model provides an invaluable model bridging murine in vivo studies and clinical trials to the study and drug development for human breast cancer

Breast cancer is the second leading cause of death in women worldwide [8]. The International Agency for Research on Cancer (IARC) estimated in 2012 that approximately 1.67 million women were diagnosed with breast cancer globally, and 522,000 died from the disease or related complications. At present, a murine model with inducible or implanted mammary tumors is used in the majority of human breast cancer studies. Despite the broad capability to investigate complex mechanisms of cancer pathways, tumor progression and therapeutic agents, genetically engineered mice possess limitations in modeling human cancer. Alterations in the immune system of certain mouse strains, inbreeding and homogeneity of experimental cells implanted into mice all lead to a limited picture of tumor biology. In this regard, the dog might be a unique alternative for breast cancer research for a number of reasons. First, the disease in dogs is pathologically similar to that in humans, and importantly, exhibits the heterogeneity that is observed in human tumors [9]. Second, the dog is one of the few species in which mammary gland neoplasms occur spontaneously with almost 3 times the incidence in humans [10]. The highest incidence occurs in intact, older bitches in the age range of 8–12 years [11]. Third, the clinical course and tumor progression of CMT is comparable to breast cancer in women. In both, for example, larger size and metastasis detected in adjacent lymph nodes or distant organs are correlated with higher tumor grade and poorer prognosis [12]. Fourth, shorter overall lifespan than human and larger size compared to mouse enables the possibility of monitoring tumor progression and treatment outcomes in a shorter period of time. Fifth, close similarity to the human genome and comparable kinetics regarding drug metabolism indicates that the canine might be an invaluable model bridging clinical trials between mouse and human (Fig. 1). Finally, environmental factors, including diet, lifestyle, and UV and chemical exposure, are similar between dogs and humans [9].

## Cancer stem cells

A solid tumor is a heterogeneous mass made up of a variety of cell types, including cancer cells, cells in the tumor microenvironment and the extracellular matrix. CSC make up a small subpopulation of the cancer cells that are thought to play a major role in tumor initiation and progression. Unique properties of the CSC, such as self-renewal, differentiation and drug resistance, determine both long-term survival as well as the ability to escape chemotherapy.

Although the initial evidence that cancer might arise from a population of stem-like cells dates as far back as the nineteenth century, improved technology in basic cancer research enabled comprehensive studies in this field [13]. The development of flow cytometry together with discovery of cell surface markers and the increased availability of xenograft mouse models for human cancer have enabled the isolation and characterization of human CSC. A study conducted by Bonnet and Dick in 1997 was the first to show that within a cancer only a certain population of cells possessed the unique potential for self-renewal [14]. They demonstrated that only the CD34^+^CD38^−^ fraction of cells isolated from human acute myeloid leukemia had the ability to initiate hematopoietic malignancy in non-obese diabetic/severe combined immunodeficiency (NOD/SCID) mice during serial transplantations. Interestingly, because of the presence of the same surface markers on normal stem cells, the authors proposed that CSC might originate from normal primitive cells rather than committed progenitor cells. Six years later, Al-Hajj et al. [15] identified cells with stem cell-like properties in solid tumors. Using the combination of anti-CD24, anti-CD44 and anti-epithelial-specific antigen (ESA) antibodies, the authors dissociated nine primary or metastatic human breast cancer samples. The cells were sorted, and 10 serial dilutions were injected into the mammary gland of NOD/SCID mice. This study revealed the explicit tumorigenic potential of selected ESA^+^CD44^+^CD24^−^/^low^ Lineage^−^ cells. As few as only 100 cells were able to initiate tumor growth. Furthermore, intra tumor heterogeneity was present in the xenografts, paralleling the cellular diversity observed in the primary counterparts. Thus, CSC not only self-renew but also differentiate into phenol typically diverse cells of a particular tumor. To date, stem-like cancer cells have been identified in numerous solid tumors including lung, prostate, brain, melanoma and ovarian cancer [16–19].

Metastasis, the development of secondary tumors in adjacent or distant organs, is estimated to be responsible for 90% of cancer related deaths [20]. It is believed to take place in part through the corruption of a normal cellular process, the epithelial-mesenchymal transition (EMT), which is observed in normal tissue development and wound healing. Reversible transformation of a polarized epithelial cell into a motile mesenchymal type enables cells to migrate to distant regions, invade tissues and generate metastases. Cells undergoing EMT are characterized by a reduced expression of E-cadherin-mediated intercellular junctions and keratin rich intermediate filaments with a concurrent increase in mesenchymal markers including vimentin and fibronectin. Recently, several studies suggested that there is a link between CSC and EMT. For instance, a study by Mani et al. [21] revealed that induction of the EMT program through SNAIL, TWIST or TGF-β1 in immortalized human mammary epithelial cells led to the generation of CD44^+^/CD24^−^ cells with stemness properties.

Treatment regimens for human breast cancer today often include chemo- and radiotherapy. However, despite these aggressive approaches, many patients experience tumor recurrence possibly due to the presence of dormant cancer cells [22]. It has been hypothesized that CSC with their multiple defense mechanisms largely make up this population of tumor cells. Chemotherapy is generally most effective against rapidly proliferating cancer cells; thus, the low proliferation rate and increased number of ATP-driven efflux transporters allow stem-like cells to escape the cytotoxic effects of multiple compounds [5]. Moreover, the over expression of anti-apoptotic proteins, such as B-cell lymphoma 2 (BCL2), and increased resistance to DNA damage make them highly resistant to radiotherapy [23]. Failure of chemo- and radiotherapy in breast cancer treatment has stimulated research for novel compounds that might selectively target those remaining CSC.

## Isolation of canine mammary cancer stem cells

**Fig. 2:**
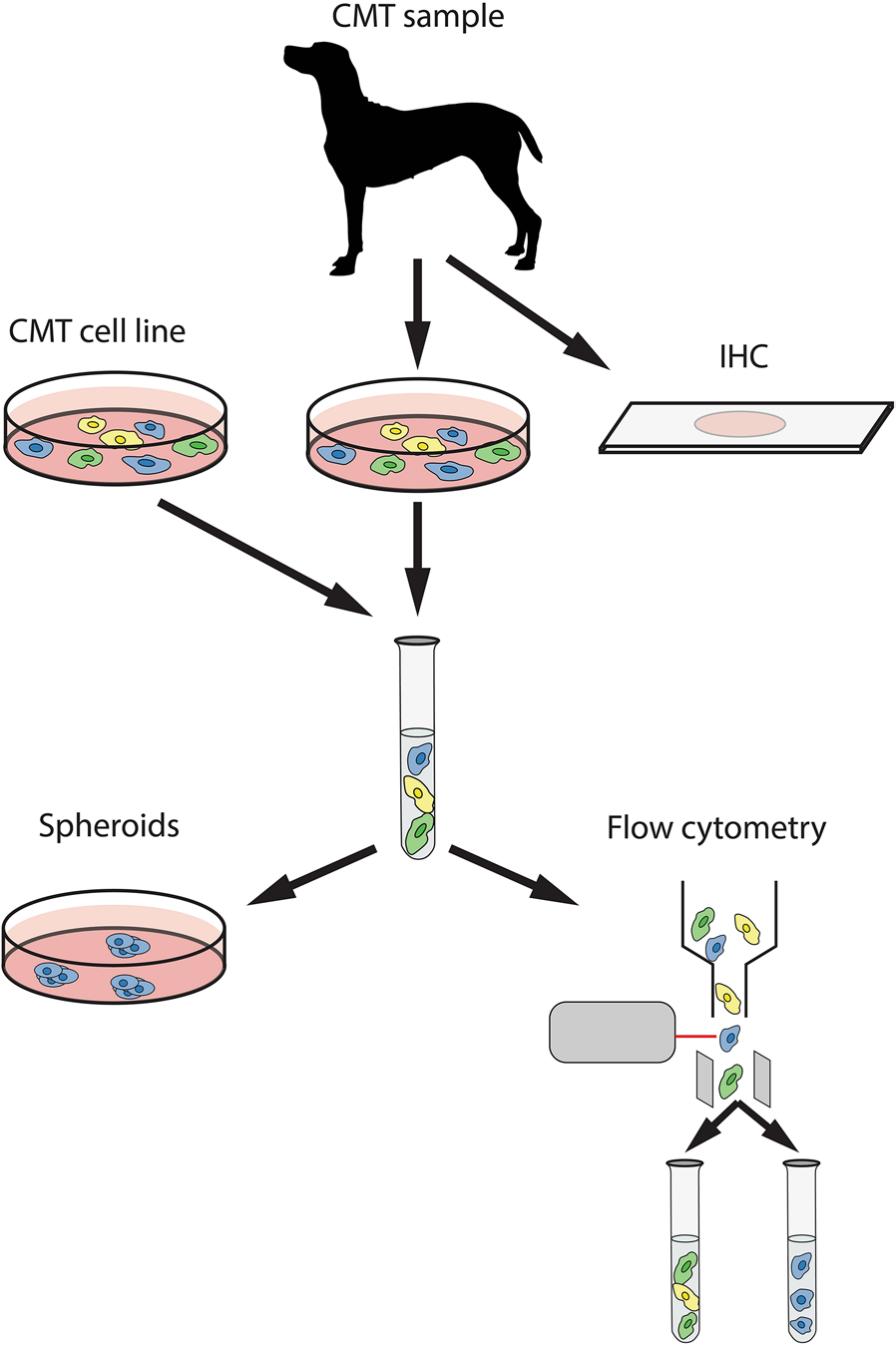
Methods used for the identification and isolation of canine mammary cancer stem cells

The discovery of CSC in human breast cancer triggered investigation of a similar subpopulation in canine mammary tumors. Based on their characteristic features, namely, sphere-forming ability under non-adherent conditions, expression of specific surface markers and high expression of ATP-binding cassette (ABC) transporters, several methods have been proposed for CSC isolation (Fig. 2).

Somatic mammalian cells have limited replicative potential and undergo cell senescence. Stem cells differ in that they possess the unique ability to self-renew and to perpetuate their pool throughout the life span of an organism. In cancer, CSC exhibit these same properties driving tumor progression and recurrence. Thus methods to enrich for more undifferentiated cell types from normal mammary gland tissue, such as the system developed by Dontu et al. [24], have been important for the functional and molecular characterization of CSC. These authors generated more favorable conditions for the proliferation of progenitor cells by culturing cells dissociated from tissue on low attachment plates in serum-free medium supplemented with B27, epidermal growth factor (EGF), basic fibroblast growth factor (bFGF) and heparin. More cells within floating spherical colonies expressed a high level of ABC transporters compared to the parental cell line (27 vs 1%, non adherent vs adherent culture, respectively). Moreover, only cells within the floating spheres were able to regenerate colonies and possessed multi lineage differentiation potential.

Using this method, Cocola et al. [25] isolated the first canine mammary CSC in 2009. Serially dissociated spheroids obtained from eight CMT samples were able to regenerate for up to 5 passages and displayed similar morphology to mammospheres obtained from normal tissue. In a 3D assay in vitro, the canine CSC cultured on collagen gels generated branching structures reminiscent of primitive tubes of the mammary gland, and when implanted into the fat pads of NOD/SCID mice, induced tumor growth. The regeneration potential as well as the number of spheroids varied in subsequent studies [25–33]. Cells isolated from the REM134 canine mammary carcinoma cell line formed spheres 16 times in long-term serial passaging. These cells were cultured under analogous conditions but were additionally supplemented with progesterone, putrescine, sodium selenite, transferrin and insulin [29]. Differences in sphere culturing media however have also been shown to influence the regeneration potential of CSC from CMT. The results of these experiments revealed that in contrast to stem cells isolated from normal mammary gland tissue, self-renewal of CSC was repressed by increased concentrations of EGF and FGF2 rather than promoted [33]. In another study, cells obtained from four canine mammary gland adenoma cell lines (1 × 10^4^) formed between 45 and 273 spheres demonstrating the existence of different percentages of progenitor cells among samples [27].

The self-renewing, sphere culturing system is a useful and easy method for the isolation of CSC; however, some limitations do exist. First, the in vitro environment may not fully reflect in vivo conditions and may lead to the selection of a specific group of cells that might vary from putative CSC. Second, calculations for sphere formation might be sometimes highly inaccurate, as so-called larger spheres are often the result of the aggregation of smaller spheres [34]. Finally, technical difficulties in obtaining single-cell suspensions might lead to biases and chimerism in the formation of spheres.

One of the most common methods currently used to isolate CSC from human breast cancer samples is flow cytometry. Based on the expression of specific cell surface markers or functional properties, CSC can be easily and quickly identified. Using multiple parameters simultaneously, flow cytometry enables the isolation of this very rare subpopulation of cells depending on set criteria. Human breast CSC have been previously characterized as CD44^+^/CD24^−/low^ [15]. Detection of CD44^+^/CD24^−/low^ cells has also been reported in CMT [27, 30, 35]. A study by Michishita et al. [27] demonstrated that 85% of cells were CD44^+^/CD24^−/low^ in spheres derived from a canine mammary adenocarcinoma cell line. Surprisingly, the authors also observed that 45.3–91.7% of cells were CD44^+^/CD24^−/low^ in the four parental cell lines analyzed. Such high levels of CD44 positive cells might however be associated with proliferation status rather than stem cell phenotype as the proportion of cells in G0/G1 that are CD44^−/low^ has been shown to be higher than in G2/M where the majority are CD44^+^ [36]. Therefore, isolation of CSCs should be performed with a combination of cell surface markers.

Ferletta et al. [30] detected canine mammary CSC using the marker stem cell antigen (Sca-1; also known as mouse mammary progenitor cell marker) and additional antibodies against CD10, CD34, CD44, CD24 and CD49f surface markers. In these experiments, the Sca-1^+^ side population from the atypical benign mixed mammary tumor line CMT-U229 expressed a CD10^low^/CD34^+^/CD44^+^/CD24^low^ and CD49f^+^ antigenic profile. Moreover, CD133^+^ cells sorted from the same Sca-1^+^ side population formed spheroids on low-attachment plates. These observations suggested that CD133 and Sca-1 might be relevant markers for canine mammary CSC isolation. Data from our own studies corroborate these results [6]. The Sca-1^+^ cells isolated from three CMT cell lines (CMT-U27, CMT-U309 and P114) formed colonies with an undifferentiated morphology and expressed CD44 and epithelial cell adhesion molecule (EpCAM) genes at significantly higher levels. However, in contrast to the results of Michishita et al. [27], only between 0.2 and 1.2% of cells in these canine mammary neoplastic cell lines were characterized with progenitor features and a Sca-1^+^ phenotype.

Immunohistochemistry (IHC) is a second method used for the identification and quantification of CSC within tumor specimens. A few groups have presented promising results using this technique [28, 37–40]. In a large study conducted by Magalhaes et al. [39], 130 CMT samples, including benign, malignant and lymph node metastases, were analyzed for CD44 and CD24 expression. The CD44^+^/CD24^−^ phenotype, which is characteristic of CSC, was detected more frequently in higher-grade tumors and lymph node metastases, while the CD24^+^ phenotype was identified more frequently in lower grade samples (grade I) compared to the other pathologies. Similar results were also demonstrated by other groups concluding that a higher frequency of CD44^+^/CD24^−^ cells is associated with poorer prognosis [37–39]. However, results might be often underestimated due to non-homogenous expression of CD44 within tissue, uneven localization of putative cells and difficulties with serial sectioning of samples [41].

Based on current knowledge, there is no single method for CSC isolation that is necessarily superior to others. The choice of sample type as well as isolation technique have certain limitations that should be considered when analyzing data. Optimization of the protocol in the future might therefore be beneficial in order to more clearly define a set of conditions that will lead to consistent and comparable results.

## Functional characterization of canine mammary cancer stem cells

Several groups have analyzed the effect of chemo- and radiotherapy on isolated canine mammary CSC. Doxorubicin is a widely used anti-tumor agent that causes DNA damage and inhibits cell proliferation. Spheres isolated from the canine mammary carcinoma cell line REM134 cultured with increasing concentrations of doxorubicin exhibited higher resistance compared to the parental, adherent cells [29]. The cytotoxic effect was almost twofold less in these cells. The doses 0.001, 0.01 and 0.05 μM left 80, 60 and 30% viable cells, respectively. The same cells were resistant to radiation doses of 1–5 Gy and exhibited a reduced loss of colony formation ability. Increased resistance to doxorubicin and no response to paclitaxel treatment were also observed by three other groups [27, 31, 35]. Barbieri et al. [31] did not observe any reduction in cell viability, even at the highest concentrations (5–10 µM) of the drugs. Discordance between results obtained using cell lines and spheres derived from tumor samples might be explained by possible different gene expressions and cellular properties that occur during the process of immortalization [24].

Recently, the effect of three alternative drugs has been investigated in the treatment of canine mammary tumors. In one study, the anti-diabetic drug metformin was shown to specifically inhibit the viability of CSC [31]. The IC_50_ concentration after treatment for 48 h varied between samples, resulting in a mean value of ~10 mM. In an in vivo study in NOD/SCID mice, a 62% reduction in tumor growth was observed in animals receiving metformin in the drinking water. In culture, only 2% of cells dissociated from tumor samples obtained from treated animals were able to form spheres compared to 13% of cells derived from the untreated group. The significantly lower clonogenic potential of CSC observed in the ex vivo assay, and the high safety of the drug make metformin a promising agent for combined anti-tumor therapy [31].

In a second study, simvastatin, a cholesterol-decreasing therapeutic used for the prevention of myocardial infarction, was tested as a potential agent to target CSC [35]. Although the drug is known to competitively inhibit 3-hydroxy-3-methylglutaryl coenzyme A reductase which is involved in cholesterol synthesis, it also reduces expression of CD44, a CSC surface marker. The effect of simvastatin on sphere forming capacity was therefore investigated in the canine mammary cancer cell line, CF41 Mg, which is enriched for CD44^+^/CD24^−/low^ cells. The number and size of colonies generated in the presence of the drug was significantly decreased in a dose-dependent manner. At the molecular level, simvastatin led to the reduction of β-catenin in the plasma membrane of cells within spheres and activation of caspase 3/7 which increased DNA fragmentation in CSC. Moreover, spheres were more sensitive to combination treatment with doxorubicin compared to spheres treated with 10 µM simvastatin alone [35].

In a third potential therapeutic strategy, melatonin, a hormone synthesized by the pineal gland, was shown to suppress properties of EMT in CSC. Under treatment with the hormone (1 mM), the expression levels of OCT4, N-cadherin and vimentin were significantly decreased in CSC isolated from the cell line CMT-U229 whereas E-cadherin levels were increased. Migration and invasion potential of treated cells was also found to be reduced, by 40% compared to controls, indicating a potential anti-metastatic role for melatonin in CMT [42].

According to Skidan and Steininger [43], anti-CSC drugs can be divided into three main categories: chemical compounds, small molecules targeting CSC and therapeutic biological agents including microRNA (miRNA) based drugs. A family of endogenous, non-coding RNAs (ncRNA) regulates ~30% of genes, and altered expression of miRNA has been associated with cancer development [44, 45]. Our group was the first to study miRNA expression in canine CSC isolated from three different CMT cell lines [6]. Comparison of miRNA profiles using a custom-designed canine miRNA microarray revealed significant deregulation of 33 miRNAs in CSC relative to differentiated tumor cells. Interestingly, genes targeted by the down regulated miRNAs were mostly involved in the TGF-β signaling pathway. Similarly, Pang et al. [29] observed that REM134 cells treated with TGF-β led to enrichment in the number of sphere-derived cells and induced EMT. Therefore, it is possible that regulation of the expression of some miRNAs might control EMT and deplete the CSC subpopulation.

It has also been recently demonstrated that non-neoplastic cell types within the tumor microenvironment influence biological properties of CSC isolated from CMT [7]. Tumor-associated macrophages (TAMs), the most abundant population of immune cells infiltrating solid tumors, have been shown to promote over expression of the monocyte chemo attractant CCL2 in CSC, which led to enhanced TAM infiltration [46]. Moreover, co-culturing with TAMs yielded CSC with higher invasive potential and elevated pro-angiogenic properties. These results corroborate previous findings associating TAMs with tumor progression and metastasis in human breast cancer [47, 48].

## Conclusions and perspectives

Studies performed in recent decades indicate that the mechanism of tumor initiation, progression and recurrence might be driven by rare subpopulation of cells called cancer stem cells. These cells possess the unique ability to self-renew and differentiate into a variety of cell types. While CSC have been previously characterized in numerous human cancers, they have only recently been identified in canine neoplasms, including mammary tumors. Interestingly, studies have demonstrated that canine mammary CSC not only express similar surface markers to human CSC but are also resistant to chemo- and radiotherapy. These results highlight the value of comparative oncology and the possible utility of the canine model in breast cancer research.

## Abbreviations

ABC: ATP binding cassette
ATP: adenosine triphosphate
BCL2: B cell lymphoma 2
bFGF: basic fibroblast growth factor
CMT: canine mammary tumor
CSC: cancer stem cells
EGF: epidermal growth factor
EMT: epithelial-mesenchymal transition
EpCAM: epithelial cell adhesion molecule
ESA: epithelial specific antigen
IHC: immunohistochemistry
NOD/SCID: non-obese diabetic/severe combined immunodeficiency
Sca-1: stem cells antigen 1
TAMs: tumor-associated macrophages
TGF-β: transforming growth factor beta
